# Long-term Autophagy and Nrf2 Signaling in the Hippocampi of Developing Mice after Carbon Ion Exposure

**DOI:** 10.1038/srep18636

**Published:** 2015-12-22

**Authors:** Fei Ye, Ting Zhao, Xiongxiong Liu, Xiaodong Jin, Xinguo Liu, Tieshan Wang, Qiang Li

**Affiliations:** 1 Institute of Modern Physics, Chinese Academy of Sciences, Lanzhou 730000, China; 2 Department of Modern Physics, Lanzhou University, Lanzhou 730000, China; 3 University of Chinese Academy of Sciences, Beijing 100049, China; 4 Key Laboratory of Heavy Ion Radiation Biology and Medicine of Chinese Academy of Sciences, Lanzhou 730000, China

## Abstract

To explore charged particle radiation-induced long-term hippocampus damage, we investigated the expression of autophagy and antioxidant Nrf2 signaling-related proteins in the mouse hippocampus after carbon ion radiation. Heads of immature female Balb/c mice were irradiated with carbon ions of different LETs at various doses. Behavioral tests were performed on the mice after maturation. Acute and chronic expression of LC3-II, p62/SQSTM1, nuclear Nrf2, activated caspase-3 and the Bax/Bcl-2 ratio were measured in the hippocampi. Secondary X-ray insult was adopted to amplify potential damages. Long-term behavioral changes were observed in high-LET carbon ion-irradiated mice. There were no differences in the rates of LC3-II induction and p62/SQSTM1 degradation compared to the control group regardless of whether the mice received the secondary X-ray insult. A high nuclear Nrf2 content and low apoptosis level in hippocampal cells subjected to secondary X-rays were observed for the mice exposed to relatively low-LET carbon ions. Therefore, carbon ion exposure in the immature mouse led to an LET-dependent behavioral change after maturation. Although autophagy was intact, the persistently high nuclear Nrf2 content in the hippocampus might account for the unchanged behavioral pattern in mice exposed to the relatively low-LET carbon ions and the subsequent increased radioresistance of the hippocampus.

Brain and central nervous system (CNS) tumors are the most common cancers in children^1^. Charged particle therapy has an established role in the treatment of head-and-neck cancers and skull base tumors^2^,^3^,^4^,^5^,^6^,^7^, especially in pediatrics^8^. However, radiation-induced brain impairments have been reported in patients after charged particle therapy^5^. Moreover, the effects of particle radiation on the central nervous system have been reported to persist for a long time^9^. Neurological complications (i.e., impairments in cognitive functioning, language acquisition, visual spatial ability, and memory and executive functioning) and changes in social behaviors were found to sometimes occur in brain tumor patients after charged particle therapy^10^. Currently, charged particle radiation-induced brain injuries in young brain tumor survivors need to be further evaluated due to the lack of clinical and experimental data^3^,^4^.

The hippocampus is a major component involved in particle radiation-induced long-term brain injury and behavioral changes^11^,^12^,^13^,^14^,^15^,^16^. A number of accelerator-based studies have noted that particle exposure leads to various hippocampus-related changes in the behavior in rodents, such as impaired spatial memory and cognitive performance^17^,^18^ and even Alzheimer’s disease-like changes^19^. The primary damage to cells in the hippocampus by ionizing radiation are DNA clustered damaged sites (made up of double-strand breaks (DSBs) with associated base lesions or abasic (AP) sites), and non-DSB clusters (comprised of base lesions, AP sites and single-strand breaks)^20^,^21^. Hudson *et al.* reported that the induction and persistence of radiation-induced DNA damage 24 hours after irradiation was more pronounced in the hippocampi of young animals than old animals^22^. Deficiency in DNA damage repair of both single-strand breaks and DSBs can lead to neurological disease^23^. Ionizing radiation-induced cognitive impairments depend on the ability to repair DNA DSBs via the NHEJ pathway^24^. Chronic inflammation and oxidative stress in the hippocampus are two major characteristics of ionizing radiation-induced neurodegenerative disorders^25^,^26^,^27^. Therefore, the autophagy pathway, which allows the degradation and recycling of damaged cellular components, and nuclear factor (erythroid-derived 2)-like 2 (Nrf2) signaling in the hippocampus are crucial defense systems against ionizing radiation. The role of autophagy in the long term effects of ionizing radiation is a controversial topic. ^56^Fe exposure has been reported to alter autophagy markers in the hippocampi of mice^28^. Poulose *et al.* reported that although the loss of autophagy occurred shortly after particle exposure, autophagy function was recovered via inhibition of mTOR in the hippocampus region of rats^29^. The transcription factor Nrf2 plays a central role against radiation-induced oxidative damage, inflammation and cell death^30^,^31^ and is a primary signaling molecule in the antioxidant system. For instance, the expression of the anti-apoptotic gene Bcl-2 is upregulated when Nrf2 migrates into the nucleus, thereby preventing cells from initiating apoptosis^32^. Furthermore, Nrf2 signaling has captured a lot of attention as a valuable therapeutic target for the treatment of neurodegenerative diseases^33^. Consequently, investigating the expression kinetics of autophagy, apoptosis and Nrf2 signaling-related proteins in heavy ion exposure-injured hippocampi of young mice can help reveal the possible mechanisms underlying the long-term effects of high linear energy transfer (LET) radiation on the brain.

In this study, the heads of 3-week-old Balb/c mice (immature stage) were irradiated with carbon ions at different LETs and doses. The mice were randomly divided into four groups for irradiation: control (CK), the high-LET and 2Gy group (LET = 70-100 keV/μm, dose = 2Gy, designated HL-2Gy), the relatively low LET and 2Gy group (LET = 10-20 keV/μm, dose = 2Gy, designated LL-2Gy), and the relatively low LET and 5Gy group (LET = 10–20 keV/μm, dose = 5Gy, indicated LL-5Gy). To observe long-term brain injuries, a battery of behavioral tests were performed at the age of 15 weeks old (mature stage). Moreover, acute and chronic responses of autophagy and Nrf2 signaling to the carbon ions were investigated by observing the expression kinetics of relevant proteins in mice with changed and unchanged behaviors after irradiation. A secondary radiation stress should induce the autophagy and Nrf2 pathway responses and the apoptosis of nerve cells. Therefore, a secondary X-ray insult was employed in this study after the behavior tests were conducted to provoke potential defects in autophagic flux and Nrf2 signaling in the carbon-ion irradiated mice. Because both autophagy^34^ and Nrf2^35^ signaling are used to modify cellular radiosensitivity, protein markers of apoptosis can be indicators of changes in autophagy and Nrf2 signaling. The proto-oncogene Bcl-2 is a founding member of the Bcl-2 family that modulates cell survival. While some members of this family (e.g., Bcl-2 and Bcl-X_L_) attenuate apoptosis, others (e.g., Bax, Bad and Bcl-X_S_) promote apoptosis^36^,^37^. Bax is believed to act by heterodimerizing with Bcl-2 through conserved Bcl-2 homology (BH) regions (B1 and B2) to antagonize its protective function^38^. Consequently, the ability of a factor to modulate the ratio of Bax to Bcl-2 is believed to be an important determinant of whether it is pro-apoptotic or anti-apoptotic^39^. In this study, the ratio of Bax to Bcl-2 was used as an indicator of neuronal cell apoptosis and brain injury. Thus, activated caspase-3 and the Bax/Bcl-2 ratio were also measured. Our study provides information that may be useful to elucidate the nature of high-LET particle radiation-induced long-term brain damage in developing mice.

## Results

### Behavior tests

#### General health status

No significant differences were observed in weight and food intake between the groups. Two weeks after carbon ion exposure, the hairs on the top of the heads of the mice within the irradiation field were lost to varying degrees depending on the radiation intensity (LET and dose), but the hairs recovered completely 1 month later.

#### Motor function

**Figure 1:**
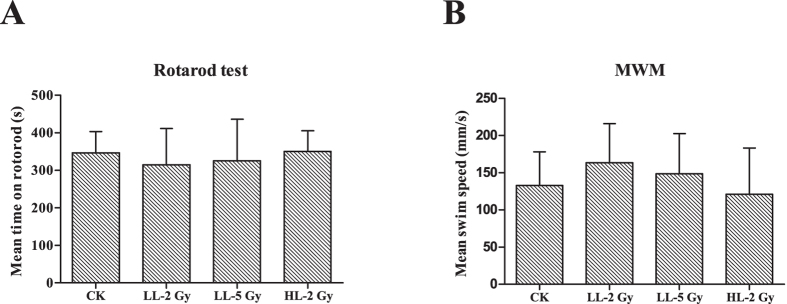
Behavior tests for motor function. (**A**) Cylinder fall latencies of the mice three months post-irradiation in the rotarod test with a rotation speed of the cylinder of 20 turns per minute. The results are presented as the mean ± standard deviation (SD) from triplicate trials. There was no difference between the groups. (**B**) Swim speed of the mice three months after irradiation in the MWM navigation trials in which the mice were allowed to swim freely for 120 s. The results are presented as the mean ± standard deviation (SD) from triplicate trials.

Motor function was estimated based on the duration on the bar in the rotarod test and the mean swim speed in the Morris water maze (MWM) navigation trials. In the rotarod test, the mean time on the bar in each group varied between 300 and 350 s (Fig. 1A). The swim speed in the navigation trials in the MWM was between 120 and 163 mm/s. No significant differences in the duration on the bar or the mean swim speed were observed between groups (Fig. 1B).

#### Spatial learning and cognition capability

**Figure 2:**
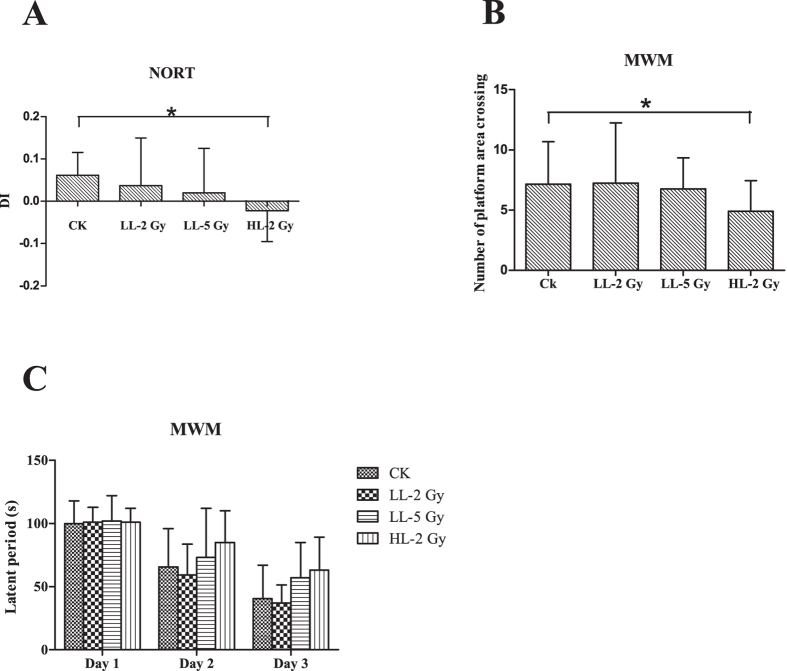
Spatial learning and cognition capability-related behavior tests. (**A**) Discrimination index of the mice in each group (CK vs. High LET 2Gy, p = 0.011). (**B**) The numbers of platform area crossings in the navigation trial of the MWM test (CK vs. High LET 2Gy, p = 0.035). (**C**) Latent period of the mice post-irradiation in the MWM test within three consecutive days. The experimental period was set to 120 s. The results are presented as the mean ± standard deviation (SD) from triplicate trials.

The object recognition ability of the mice was measured using the novel object recognition task (NORT) and is shown in Fig. 2A. Significant cognitive deficits were found in the mice in the HL-2Gy group compared to the CK. All discrimination indices (DI) were greater than zero with the exception of the HL-2Gy group (DI_CK_ = 0.06, DI_LL-2Gy_ = 0.004, DI_LL-5Gy_ = 0.02, and DI_HL-2Gy_ = -0.02; CK vs. LL-2Gy, *p* = 0.070; CK vs. LL-5Gy, *p* = 0.179; and CK vs. HL-2Gy, *p* = 0.011). The spatial learning and memory capabilities of the mice were evaluated using the MWM test. The latent periods in all groups gradually shortened over three continuous days, from 99.8s to 40.4s in CK, 101.3s to 37.4s in the LL-2Gy group, 102.2s to 57.0s in the LL-5Gy group, and 101.8s to 63.4s in the HL-2Gy group. No difference was observed in the latency of the ability of the mice to find the hidden platform on three successive days (Fig. 2C) (CK vs. LL-2Gy, *p* = 0.773; CK vs. LL-5Gy, *p* = 0.546; and CK vs. HL-2Gy, *p* = 0.610). However, there was a severe performance decrement in the number of platform area crossings in the navigation trials for the mice in the HL-2Gy group. The mean times of the platform area crossing in the LL-2Gy, LL-5Gy, HL-2Gy and CK groups were 7.25 ± 4.99s, 6.75 ± 2.6s, 4.9 ± 2.6s and 7.15 ± 3.53s, respectively (Fig. 2B) (CK vs. LL-2Gy, *p* = 0.824; CK vs. LL-5Gy, *p* = 0.503; and CK vs. HL-2Gy, *p* = 0.035).

#### Depression-related behaviors

**Figure 3:**
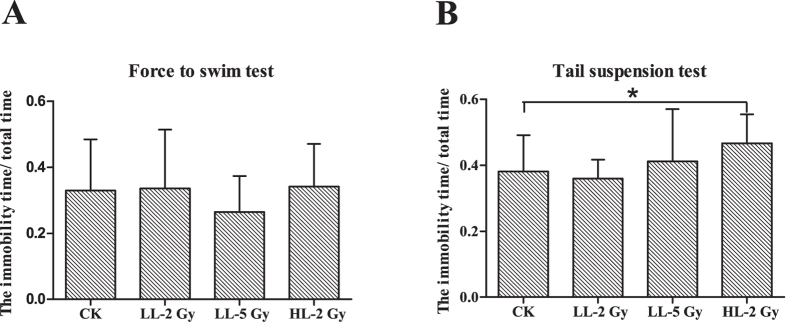
Depression-related behavior tests. (**A**) The ratio of immobile time to total time in the forced swim test. (**B**) The ratio of immobile time to total time in the tail suspension test.

Depression is a major concern for radiation-induced mood changes. None of the treatments had an effect on the immobility duration in the forced swim test compared to CK (Fig. 3A). The percentage of the immobile time out of the total time for all groups was greater than 25%. Conversely, the tail suspension test showed that the immobility duration increased in the HL-2Gy group compared to CK (Fig. 3B). The percentage of the immobile time increased significantly in the HL-2Gy group (46.6 ± 8.7%) compared to the CK group (38.0 ± 11.0%). The percentage of the immobile time was 35.9 ± 5.7% in the LL-2Gy and 41.1 ± 15.8% in the LL-5Gy groups (CK vs. LL-2Gy, *p* = 0.329; CK vs. LL-5Gy, *p* = 0.072; and CK vs. HL-2Gy, *p* = 0.034).

### Kinetics of the expression of apoptosis, autophagy and antioxidant-related proteins

#### Acute response

**Figure 4:**
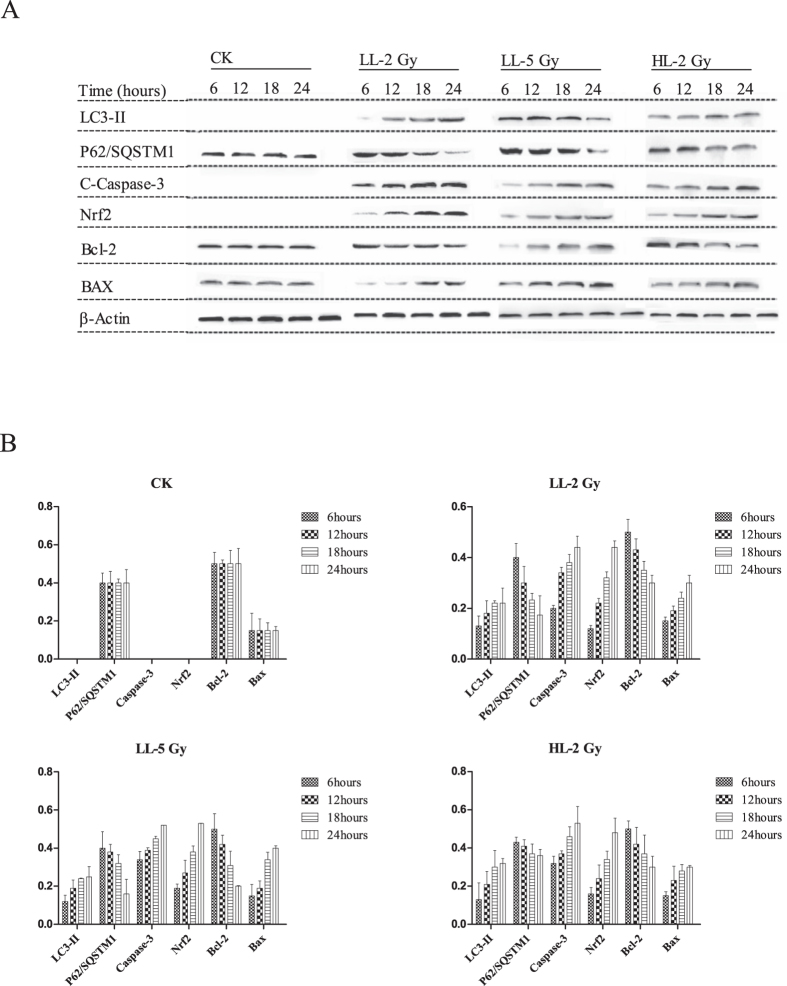
(**A**) Western blot analysis for the acute response of proteins related to apoptosis, autophagy and Nrf2 signaling after carbon ion irradiation. Hippocampi were isolated within two days after irradiation and total proteins were extracted. (**B**) The protein content normalized with the β-actin content is shown in the histogram, where the ordinate *y*-axis represents the expression ratio of target protein to β-actin.

The results of the western blot analyses for investigation of the acute response of the mice are shown in Fig. 4. Clearly, autophagy was induced in all groups based on the accumulation of LC3-II as soon as 6 hours after the carbon ion irradiation, and the autophagic levels increased over time. The expression of LC3-II was higher in the LL-5Gy group than in the other groups at each time point. The increasing trend of LC3-II expression ceased at 18 hours. The expression of p62/SQSTM1 exhibited a reducing trend. The expression of active caspase-3 was slightly increased from 6 hours in each group and was continuously observed within 24 hours. Compared to the other groups, the apoptotic level was higher at each time point and the Bax/Bcl-2 ratio was changed from 0.3 ± 0.04 to 2.0 ± 0.06 in the LL-5Gy group. The Bax/Bcl-2 ratio increased slowly and obtained a steady status at 18 hours (0.3 ± 0.05 to 1.0 ± 0.04 in the LL-2Gy group and 0.3 ± 0.08 to 1.0 ± 0.02 in the HL-2Gy group). With regards to the antioxidant response, Nrf2 increased in the nuclei in all irradiated mice in all groups a few hours after carbon ion exposure.

#### Chronic response

**Figure 5:**
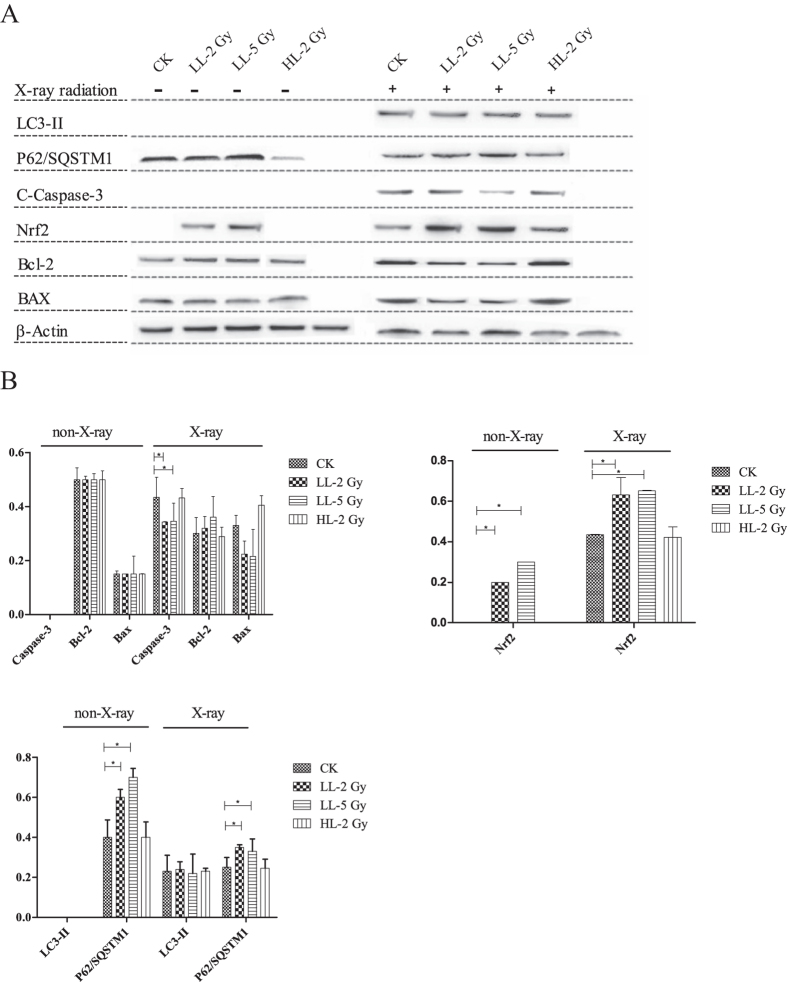
(**A**) Western blot analysis for the chronic response of proteins related to apoptosis, autophagy and Nrf2 signaling post-irradiation. The activated caspase-3, Bcl-2 and Bax (**B**), nuclear Nrf2 (**C**), LC3-II and P62/SQSTM1 content (**D**) normalized with the β-actin content is shown in the histogram, where the ordinate Y-axis represents the expression ratio of target protein toβ-actin.

Fourteen weeks after carbon ion exposure, half of the animals in each group were irradiated with X-rays a second time. All animals were euthanized 12 hours after the X-ray insult. The results of the western blot analyses for the mice under investigation for chronic responses are shown in Fig. 5. LC3-II and active caspase-3 were absent in all groups that did not undergo a second round of X-ray irradiation. The expression of p62/SQSTM1 was higher in the LL-2Gy and LL-5Gy groups, and nuclear Nrf2 was present only in these groups (LL-2Gy vs. CK, *p* = 0.0074; and LL-5Gy vs. CK, *p* = 0.0042). The Bax/Bcl-2 ratio was approximately 0.3 in all groups. LC3-II expression was equally induced in each group of mice irradiated with the X-rays, and the expression of p62/SQSTM1 was higher in the LL-2Gy and LL-5Gy groups. The Bax/Bcl-2 ratios were 1.1 ± 0.02, 1.4 ± 0.05, 0.7 ± 0.02 and 0.6 ± 0.03 in the CK, HL-2Gy, LL-2Gy and LL-5Gy groups, respectively. The expression trend of active caspase-3 was similar to the Bax/Bcl-2 ratios and was higher in the HL-2Gy and CK groups compared to the other groups. The nuclear Nrf2 content was significantly higher in the LL-2Gy and LL-5Gy groups (LL-2Gy vs. CK, *p* = 0.0482; and LL-5Gy vs. CK, *p* = 0.0032).

## Discussion

Charged particle exposure causes brain impairments during particle therapy against pediatric brain tumors. Mounting evidence suggests that charged particles result in long-term hippocampus-related functional deficits in young brain tumor survivors. Oxidative stress and autophagy are large concerns. Therefore, autophagy and anti-oxidative Nrf2 signaling in the hippocampi of developing mice under particle radiation stress needs to be carefully investigated.

An accurate particle radiation system for mouse head exposure was established in this work. The heads of immature female Balb/c mice (3 weeks old) were irradiated with carbon ions of different LETs. After irradiation, behavioral changes in the mature mice (15 weeks old) were evaluated using various behavior tests. The behavior tests showed that the high-LET carbon ions led to a long-term behavioral pattern disorder in the mice that received the 2Gy dose, whereas the relatively low-LET carbon ions at the 2Gy and 5Gy doses did not cause behavioral pattern changes a long time after irradiation, indicating that the behavior changes occurred in an LET-dependent manner. These results are consistent with the finding by Rabin *et al.*^40^, who showed that the effectiveness of the long-term cognitive performance decrement in rodents was directly proportional to the LET value of the carbon ions. Other factors that influence the effectiveness and cannot be ignored are age^41^ and sex^42^. Indeed, the high-LET carbon ions in this study elicited long-term brain injury in mice after irradiation based on the decrement in behavioral performance.

In the acute response, autophagy, apoptosis and Nrf2 signaling were all activated in the hippocampus within a few hours post-irradiation. Both the levels of autophagy and apoptosis increased with the radiation intensity (dose and LET), suggesting that autophagy and apoptosis could be induced by carbon ions simultaneously in a dose- and LET-dependent manner. It has been reported that Nrf2 is usually observed in the nuclei of hippocampal neurons^43^. However, nuclear Nrf2 was absent in the CK group in this study, possibly because Nrf2 was barely present in the nuclei of the glial cells that predominated in the brain cells^43^.

Autophagy dysfunction has been observed in various neurodegenerative diseases^44^, such as the p62/SQSTM1 accumulation found in neurofibrillary tangles in Alzheimer’s disease^45^. However, whether the autophagic flux is influenced a long time after exposure to high LET radiation is largely unknown. In the chronic response in this study, the induced LC3-II content in all secondary X-ray insulted groups was identical, suggesting that the initiation stage of autophagy was not disturbed by the carbon ion exposure. Interestingly, the amount of p62/SQSTM1 proteins was higher in the LL-2Gy and LL-5Gy groups than in the CK group regardless of whether the mice received the secondary X-ray insult, indicating that the irradiation with carbon ions at a relatively low LET (2Gy and 5Gy) restrained the p62/SQSTM1 degradation-related process at the autophagosome clearance stage (i.e., autophagosome fusion with the lysosome). However, Jain *et al.* suggested that p62/SQSTM1 was a target gene for Nrf2 and that the expression of p62/SQSTM1 increased when Nrf2 amassed in the nucleus^46^. Moreover, as shown in Fig. 5 using the group without secondary X-ray insult as the reference, the degradation rates of p62/SQSTM1 in the LL-2Gy and LL-5Gy groups were approximately equal to or slightly higher than the rates in the CK group 12 h after the secondary X-ray insult (from 0.60 to 0.35 in the LL-2Gy group, from 0.70 to 0.33 in the LL-5Gy group, and from 0.40 to 0.25 in the CK group). Furthermore, the animals in the LL-2Gy and LL-5Gy groups performed normally in the behavior tests. Therefore, we could deduce that the nuclear Nrf2 accumulation was responsible for the increase in p62/SQSTM1 observed in the LL-2Gy and LL-5Gy groups and that the autophagy flux was not influenced by the initial carbon ion exposure.

Unexpectedly, the Nrf2 content in the nucleus in the LL-2Gy and LL-5Gy groups was obviously higher 14 weeks post-irradiation compared to the CK and HL-2Gy groups. The reason for this phenomenon requires further investigation. Regardless, there might be two possible directions for future studies. First, Nrf2 signaling is evoked by the oxidative stress and chronic inflammation that are features of radiation-induced long-term brain injury and leads to persistent ROS accumulation. The inflammatory process in the brain is not directly proportional to the radiation dose two months after iron particle exposure^47^, and low dose radiation activates more microglia in the hippocampal dentate gyrus. Therefore, the nuclear Nrf2 content is not necessarily consistent with the intensity of the initial carbon ion exposure. Second, different levels of oxidative stress are needed to induce Nrf2 signaling activation in different cell types. For example, Bell *et al.* reported that subtoxic doses of H_2_O_2_ failed to activate Nrf2 signaling in astrocytes, whereas mild oxidative insults strongly elicited astrocytic Nrf2-dependent gene expression^48^. Different radiation qualities and doses cause different levels of oxidative stress in the mouse brain, possibly leading to Nrf2 signaling activation in specific types of brain cells. Because different types of brain cells account for different proportions, nuclear Nrf2 may not be detected in pooled protein samples.

The secondary X-ray exposure was expected to evoke the potential damage induced by the initial carbon ion irradiation. Surprisingly, it resulted in the decreased expression of activated caspase-3 and the reduced ratio of Bax/Bcl-2 in the LL-2Gy and LL-5Gy groups compared to the CK group. This phenomenon resembles the radiation-induced adaptive response (RAR) in which a low adapting dose of radiation can increase cellular resistance against a subsequent challenging radiation. RAR has been observed at low doses and for short time intervals between two sequential radiations both in intro and *in vivo*, such as cultured glia from the hippocampi of Wistar rats^49^ and the dentate gyrus from rats^50^. Moreover, hippocampal RAR has been observed in young rats but not aged and fetal rats^51^,^52^. Different from RAR, our findings occurred a long time after the initial exposure, and both sequential irradiations were performed at high doses. Therefore, the mechanisms underlying our RAR-like observation and real RAR would be totally different. In this study, the RAR-like finding was observed in the LL-2Gy and LL-5Gy groups in which we observed the activation of Nrf2 signaling and unchanged behavior patterns of the mice. Nuclear Nrf2 has been reported to be beneficial for neurodegenerative diseases^53^,^54^,^55^ and to contribute to RAR under the proper conditions^56^. Therefore, the activated Nrf2 antioxidant response might be responsible for the RAR-like phenomenon observed in this study.

The present study demonstrated that exposure of the immature mouse hippocampus to carbon ions caused an LET-dependent behavior pattern change after maturation and an LET-dependent differential molecular response. Carbon ions with higher LET values more easily induced the reduced performance of mice in the behavior tasks. Autophagy was intact in the hippocampal cells of the mature mice after irradiation. The persistently high nuclear Nrf2 content in hippocampal cells might account for the unchanged behavioral patterns of mice after exposure to relatively low LET carbon ions and the subsequent increased radioresistance of the hippocampal cells.

## Materials and Methods

### Animals

One hundred and twenty 3-week-old female Balb/c mice were used in this study. The mice were housed in a well-designed facility with 12 h light/dark cycles and access to food and water ad libitum at the Institute of Modern Physics (IMP), Chinese Academy of Sciences. All experiments were conducted in accordance with the requirements of the Animal Care Committee and with the approval of the Academic Committee at IMP. According to The Jackson Laboratory, mice reach maturity at 3 to 6 months, and maturity is the reference for any age-related changes (http://research.jax.org/faculty/harrison/ger1vLifespan1.html#VD). In this study, mice were irradiated at an age of three weeks (immature stage), and long-term behavioral and molecular tests were performed in mice at 15 and 17 weeks of age (mature stage).

### Irradiation

**Figure 6:**
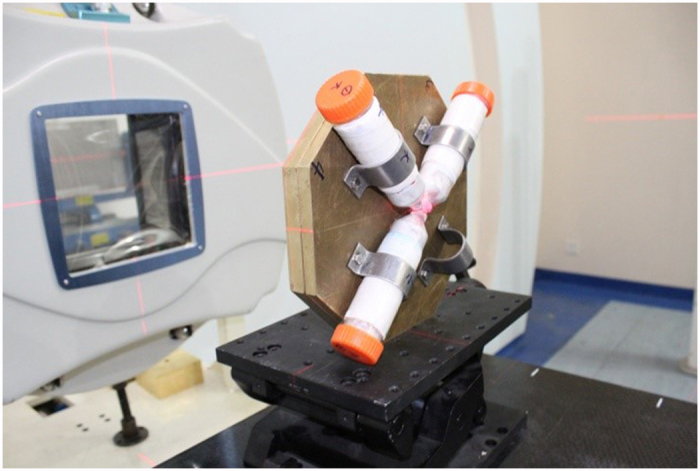
The experimental setup used in this study. The heads of the mice were irradiated with carbon ions behind a brass collimator with a 5-cm thickness.

Mice (n = 30/group) were exposed to a carbon ion beam (165 MeV/u) generated by the Heavy Ion Research Facility in Lanzhou (HIRFL) at IMP under two different conditions. First, mice brains were irradiated with carbon ions when they traversed the heads of the mice. Therefore, the doses delivered to the brains were caused by the carbon ions with a relatively low linear energy transfer (LET) (10-20 keV/μm). Second, a mini-ridge filter was inserted into the beam line so that the carbon ion beam presented a Gaussian-shaped spread-out Bragg peak (SOBP) with a full width at the half maximum (FWHM) of 5 mm in the depth-dose distribution. The depth-dose distribution of the SOBP beam was calculated using the method provided by Yousuke *et al.*^57^. During irradiation, an energy degrader in the beam line was used to adjust the range of the SOBP carbon-ion beam to localize the SOBP in the mouse brains. In this case, doses in the mouse brains were deposited by carbon ions with dose-averaged LETs of 70-100 keV/μm. Under both conditions, the depth-dose and dose-averaged LET distributions of the carbon ion beam were calculated with the one-dimensional particle and heavy ion Bragg curve calculator (HIBRAC). HIBRAC is a very well benchmarked and validated one-dimensional deterministic particle and a heavy ion computer code designed, developed and optimized by Sihver *et al.*^58^,^59^. The intensity of the carbon ion beam was adjusted to deliver doses at a dose rate of approximately 0.5Gy/min under the two irradiation conditions. In both cases, the mice were restrained using well-ventilated plastic tubes that were placed perpendicular to the beam direction and positioned so that the heads of the mice were aligned with the beam line during irradiation. A brass collimator (25-mm thick) with four apertures (10 mm in diameter each) was used to shield the bodies of the mice and confine the radiation exposure only to the heads of the subjects. The carbon ion beam was laterally scanned in a continuous zig-zag pattern by a pair of orthogonal dipole magnets upstream of the brass collimator to generate a uniform irradiation field of 50 mm×50 mm. When the brass collimator was set in the beam line, the lateral dose uniformity of the irradiation fields behind the four apertures of the collimator was measured with GAFchromic EBT film to be close to 100%, while the shielding effect of the other part of the collimator was perfect. Four mice at most could be irradiated at the same time. The center of the aperture was approximately aligned with the bregma zero coordinate of a mouse so that the mouse hippocampus was in the particle radiation field. A photograph of the irradiation unit including the brass collimator and three fixed mice is shown in Fig. 6.

**Figure 7:**
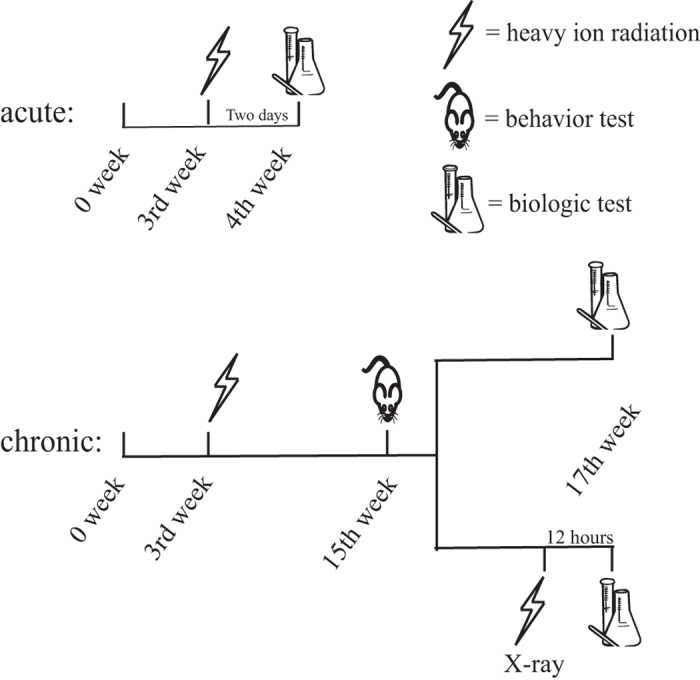
Schematic drawing of the experimental design. The sample sizes were 10 for the behavior tests and 5 for biological measurement.

The mice in the CK group were also restrained in plastic tubes for several minutes and sham-irradiated at the irradiation site. To observe the acute radiation responses, two-thirds of the animals in each group were euthanized for hippocampus separation within two days after carbon ion irradiation (6, 12, 24 and 48 hours, n = 5/time point). The remainder of the mice in each group were subjected to various behavior tests at the 12th week after irradiation in order to acquire their behavior patterns. To evoke the potential damage induced by the initial carbon ion irradiation, half of the mice in each group (n = 5) were irradiated with X-rays (50kVp) at a dose of 10Gy after the completion of the behavior tests. Twelve hours after the X-ray exposure, all of the mice were euthanized for the separation of their hippocampi. The experimental scheme of this study is schematically illustrated in Fig. 7.

### Behavior tests

The purpose of the behavior tests was to detect the mice with long-term brain injuries. Therefore, the behavior tests (n = 10/group) were performed during the 12th week after carbon ion irradiation during the period of 10:00 am to 04:00 pm each day (at 15 weeks old). Behavioral parameters were recorded, elaborated and subsequently analyzed by an automated video tracking and activity monitoring system. The apparatuses used for the behavior tests (i.e., MWM, rotarod, tail suspension test, and NORT) were purchased from Shanghai Xinruan Information Technology Co. Ltd. (China).

#### Morris water maze

Water maze tests were performed in a black bottom circular pool (120 cm in diameter) filled with water (19 ± 1 °C) to a depth of 25 cm. The water maze was divided into four quadrants and placed in a quiet room decorated with visual cues. To avoid overwhelming the possible differences between groups due to full training, the tests were performed only on three consecutive days. The platform (10 cm in diameter) was above the water surface on the first day and submerged 0.5 cm below the water on the second and third days. On the first day, the mice were placed into the maze at different starting positions facing the pool wall. Each mouse was given 120 s to look for the visible platform. If a mouse failed to find the platform within 120 s, it was guided onto the platform with a stick. Then, the mouse was allowed to stay on the platform for 30 s to familiarize itself with the location of the platform relative to the visual clues. On the third day, 1 hour after the third training trial, the platform was removed from the pool. Then, the mice were allowed to swim freely for 120 s to perform navigation trials. The latent period in the routine MWM test and times of platform area crossing in the navigation trials were recorded for analysis.

#### Rotarod test

Mice were placed on a rotating cylinder 6 cm in length and 3.5 cm in diameter in a rotarod apparatus (YLS-4C, Biowill, China). The rotation speed of the cylinder was 20 turns per minute. The heads of the mice were orientated opposite to the rotation direction. If a mouse stayed on the rotating cylinder or actively left the apparatus within 900 s, the trial was stopped. The cylinder fall latencies were measured, and then the mean fall latency of three trials was calculated.

#### Forced swim test

Mice were forced to swim in an open cylindrical container (30 cm in diameter and 60 cm in height) containing water 40 cm in depth at 19 ± 1 °C. During the test, the times taken for climbing, swimming and immobility were measured during a 6-min period. Climbing behavior consisted of upward directed movements of the mouse forepaws along the side of the swim chamber. Swimming behavior was defined as movements (usually in a horizontal direction) throughout the swim chamber. Immobility was assigned when a mouse stopped struggling or floated with only slight arm and leg action while keeping its head above water.

#### Tail suspension test

Mice were suspended by their tails for 6 minutes. During the time, escape-related behaviors of the mice, such as active movement followed by immobility, were assessed. The times spent for different behaviors (struggling and immobility) within the 6 minutes were recorded.

#### Novel object recognition task

The novel object recognition test was performed in a square test arena (40 cm side lengths) with surrounding walls 40 cm in height. All animals were given a habituation session in which they were allowed to explore the apparatus (without objects) for 5 min. On the object recognition trial, a mouse was placed into the arena with two identical objects (A and B) and allowed to explore for 5 min (training session). One hour after the training session, object B was replaced with a novel object (C). The time required for the mouse to explore each object was recorded. The DI is defined as: DI = (T_C_ − T_A_)/(T_A_ + T_C_), where T_A_ and T_C_ are the times required for the object A and C explorations, respectively. Object exploration is defined as directing the nose to the object at a distance of less than 2 cm.

### Western blot analysis

To investigate the acute and chronic responses of the autophagy and antioxidant systems to carbon ions, hippocampi were harvested within 24 hours and 14 weeks post-irradiation, respectively. Briefly, hippocampi were isolated after perfusion, cut into small pieces (approximately 1 mm^3^) and then homogenized in pre-cooled RIPA buffer with protease inhibitors according to the manufacturer’s instructions (BestBio, China). For identification of the entire cellular pool of p62/SQSTM1, an improved lysis buffer was used (1% SDS, 1 mM EDTA, 50 mM NaCl and 100 mM Tris-HCl, pH 7.4). To extract Nrf2 from the nucleus, a part of the homogenate was treated with a nuclear protein extraction kit (Beyotime Biotechnology, Wuhan, China). The concentration of proteins was determined with the BCA Protein Assay Kit (Pierce, Rockford, IL, USA). A total of 20 μg of proteins were loaded in each well on a sodium dodecylsulfate-polyacrylamide gel. The proteins on the gels were transferred to polyvinylidene difluoride membranes after electrophoresis. After blocking with 5% non-fat dry milk for 4 hours, the membranes were incubated overnight at 4 °C with primary antibodies, including rabbit monoclonal anti-caspase-3, anti-p62/SQSTM1, anti-LC3, anti-Bax, anti-Bcl2 and anti-β-actin (1:1000; Cell Signaling Technology). Then, the membranes were washed with Tris-buffered saline-Tween-20 (3 × 5 min) and incubated with the secondary goat anti-rabbit IgG-horseradish peroxidase antibody (Bioworld; 1:5,000) for 2 hours. Protein bands were visualized using the ECL chemiluminescent HRP substrate (Bioworld). Relative optical densities were subsequently measured using a chemiluminescent imaging system (Tanon 4200SF, China). The western blot measurements were conducted for each individual animal sample. For comparison purposes, all of the data of the relative optical densities were normalized to the internal control (β-actin from a unified sample) on each membrane.

### Statistical analysis

The data are expressed as the mean ± standard deviation (SD). Escape latency differences between groups in the Morris water maze tests measured on three successive days were analyzed using a two-way analysis of variance (ANOVA) with repeated measurements with two factors (group and training day). Multivariate tests were performed at the level of *p* < 0.05 in Mauchly’s test. The other data were analyzed using a one-way ANOVA analysis followed by Tukey’s test. *p* < 0.05 was considered statistically significant. The data were analyzed using SPSS 16.0 software (SPSS, Chicago, IL, USA).

## Additional Information

**How to cite this article**: Ye, F. *et al.* Long-term Autophagy and Nrf2 Signaling in the Hippocampi of Developing Mice after Carbon Ion Exposure. *Sci. Rep.* **5**, 18636; doi: 10.1038/srep18636 (2015).
